# Analysis of Radon Measurements in Relation to Daily Seismic Activity Rates in the Vrancea Region, Romania

**DOI:** 10.3390/s22114160

**Published:** 2022-05-30

**Authors:** Juan José Galiana-Merino, Sergio Molina, Alireza Kharazian, Victorin-Emilian Toader, Iren-Adelina Moldovan, Igor Gómez

**Affiliations:** 1 Department of Physics, Systems Engineering and Signal Theory, University of Alicante, Crta. San Vicente del Raspeig, s/n, 03080 Alicante, Spain; 2 University Institute of Physics Applied to Sciences and Technologies, University of Alicante, Crta. San Vicente del Raspeig, s/n, 03080 Alicante, Spain; 3 Department of Applied Physics, University of Alicante, Crta. San Vicente del Raspeig, s/n, 03080 Alicante, Spain; sergio.molina@ua.es (S.M.); igor.gomez@ua.es (I.G.); 4 Multidisciplinary Institute for Environmental Studies, University of Alicante, Crta. San Vicente del Raspeig, s/n, 03080 Alicante, Spain; alireza.kharazian@gcloud.ua.es; 5 National Institute for Earth Physics, Calugareni 12, RO 077125 Magurele, Romania; victorin@infp.ro (V.-E.T.); iren@infp.ro (I.-A.M.)

**Keywords:** radon measurements, Gutenberg–Richter distribution, daily seismic activity rate, discrete wavelet transform

## Abstract

Many previous research studies have shown how local and even regional earthquakes can significantly affect the release of radon in the soil. The aim of this work is to investigate the relationship between radon measurements and the daily seismic activity rate and develop a methodology that allows estimating the seismic activity rate using only radon measurements. To carry out this study, the earthquake catalogue of the Vrancea region (Romania) has been used to estimate the daily seismic activity rate during a given time period, in which radon measurements were also recorded, from January 2016 to September 2020. The Vrancea zone represents the most active seismic zone in Europe and is located on the eastern edge of the strongly bent Carpathian arc. In the case of the radon measurements, seasonal behaviours and linear trends due to non-seismic factors have been identified and subsequently removed. The discrete wavelet transform has been used to analyse the radon signal at two different scales: long and short periods. From the analysis carried out on a long-period scale, an approximate linear relationship has been obtained between the radon series and the daily seismic activity rate, which provides insights into the behaviour of the seismic activity in the study region with only the radon information. In addition, the study reveals certain characteristics that could be used as precursors of earthquakes at different scales: weeks in the case of the estimated daily seismic activity rate, and days in the case of the short-period signal obtained by the wavelet analysis. The results obtained for this region allow us to hope that the analysis of the radon time series can become an effective complement to the conventional seismic analysis used in operational earthquake forecasting.

## 1. Introduction

The release of radon from natural minerals has been known since the 1920s but its monitoring has more recently been used as a possible tool for earthquake prediction because the distribution of the soil–gas radon concentration is closely related to the geological structure, fracture, nature of rocks, and distribution of sources [1]. Based on radon anomalies observed before earthquakes, most researches [2,3,4] have indicated that changes in the radon release rate may be one of the key earthquake precursory phenomena. ^222^Rn is believed to emanate from fractures in the rocks and soil, an inherently porous medium [1].

The main radon isotope ^222^Rn in groundwater and near-surface air is generated by the alpha decay of the radium isotope ^226^Ra as part of the uranium decay chain [5]. ^222^Rn has a half-life of 3.8 days. Radon is retained by triple-bonded carbon compounds in the soil and released when these carbon triple bonds are oxidized to double bonds through positive hole charge carriers, which are activated by local and regional tectonic stresses. In addition, these carriers present a high capability to spread over large distances and are prone to accumulate at the surface [5].

Thus, it is to be expected that local earthquakes and even regional ones can significantly affect the activation of these carriers and, therefore, the release of radon in the soil. As a result, deviations (or anomalies) from the mean (seasonal) radon behaviour can be related to changes in seismic activity [6]. Regarding the anomalies, it is important to take into consideration the following issues: (a) the identification of the maximum distance between the epicentre of an earthquake and the site where the anomaly of radon is observed [6]; (b) the period between the observation of the anomaly and the occurrence of an earthquake of a given magnitude (precursor time) and the duration of the anomaly after the earthquake [7]; (c) the magnitude of the upcoming earthquake and the importance of the tectonic structure of the seismogenic source [6].

In this sense, several research groups have investigated the relationship between the magnitude M and the distance D between the epicentre of an earthquake and the site of the observed geophysical or geochemical anomaly. Riggio and Santulin [6] reviewed the current literature pointing out that a widely used relationship for radon diffusion is that of Hauksson and Goddard [4]: M = 2.4 log10 D − 0.4, where M is the minimum magnitude required to obtain a radon anomaly at distance D (km).

Nevinsky et al. [8] developed relationships between magnitude M and distance D (km) using soil and groundwater radon from recording stations around the Black Sea and the results were compared with previous relationships using the same stations. Attending to their results, a distance D ≤ 10 km is needed to obtain a radon anomaly from a minimum magnitude of 2.8 ± 1.0 (mean value from all the formulas analysed) and a distance D ≤ 70 km from a minimum magnitude of 4.0 ± 0.7. In addition, the formulas indicate that earthquakes with a minimum magnitude of 5.6 ± 0.9 at a distance D ≤ 1000 km may also be responsible for anomalies in the radon recording.

More recently, Papachristodoulou et al. [9] applied the relation R_E_ ≤ 1.5 × 10^0.43M^ in northwestern Greece to constrain the earthquake catalogue that could generate soil radon anomalies at the monitoring site. Concretely, R_E_ is the distance between the epicentre and the measuring site.

The application of any of these relations allows making a selection of the used catalogue events and can give information on the area affected by the deformation process that precedes an earthquake of a given magnitude, defining the distance at which a radon anomaly is attributable to a given earthquake might be detectable.

According to these studies, strong earthquakes may be correlated with radon anomalies within a large distance from the epicentre to the location of the radon station. However, in the case of persistent low-amplitude seismicity, the possible correspondence with radon anomalies is more ambiguous. This may explain why it is so difficult to relate a specific radon anomaly with a given earthquake. Radon recordings contain information on radon anomalies due to many earthquakes (seismic activity) in the area of influence determined by the distance D (which increases with the size of the earthquake). Therefore, these radon anomalies will be recorded in the station for larger magnitudes, but they will be incomplete for lower magnitudes because many of those smaller earthquakes will be outside of the area of distance D.

On the other hand, the literature indicates that the precursor time used to be between 2 days and 3 weeks before the occurrence of an earthquake, although anomalies occurring 45 days before the occurrence of large earthquakes [10] and even 485 days before the occurrence of a 7.2 magnitude in China [1] have been reported. In conclusion, neither the start nor the end of a radon anomaly seems to be directly related to the origin time of the imminent earthquake, although often an earthquake occurs within a month of an increase in radon emissions and the anomaly can continue after the event.

Regarding the connection between the magnitude of the upcoming earthquake and the corresponding radon anomaly, Walia et al. [11] proposed two linear relationships between the logarithm of the product of radon anomaly and epicentral distance and the magnitude of the earthquakes due to the different behaviours of small earthquakes (M ≤ 3.5) and moderate to large earthquake (M > 3.5).

In this way, different rules to identify earthquakes relating to radon signals have been proposed by several researchers [3,8,12,13,14,15,16]. Most of them are based on the detection of radon anomalies, defined as a positive deviation that exceeds the mean radon level by more than twice the standard deviation.

However, according to Cicerone et al. [1], although there is a very wide range of earthquake magnitudes for which anomalous radon precursors have been reported, there is still no consensus on a direct relationship between the radon anomalies and the magnitude of the upcoming earthquake. Most of the observed radon changes (50% to 100% change relative to the background radon level) are for earthquakes greater than magnitude 4.0 and usually, the reported change is an increase in the radon concentration prior to the earthquake [1].

From the previous discussions, we assume the hypothesis that changes in the radon release rate are not produced by a single large earthquake, but are a cumulative effect due to the seismicity in the area of influence of the recording station. This cumulative radon anomaly behaviour might be assumed to be similar to the Gutenberg–Richter (GR) distribution of seismicity [17,18] in the influence area of the station, also showing a magnitude of completeness related to the size of the area of influence and the fact that the radon anomalies due to small magnitudes at a long distance from the stations are not recorded.

From the viewpoint of operational earthquake forecasting (OEF), a time-dependent probabilistic seismic hazard analysis (TD-PSHA), based not only on the temporal changes of the seismicity but also on the temporal changes of the radon measurements in a seismic region, will increase the accuracy of the results. The frequency of earthquakes generated by any seismic source defines the earthquake recurrence model usually used in probabilistic seismic hazard analysis (PSHA). Gutenberg and Richter [17,18] proposed the simplest and widely used earthquake recurrence relationship as given in Equation (1).

(1) $$\log_{10}\left(\lambda_{M}\right)=a-b\cdot M$$

where $\lambda_{M}$ is defined as the total number of earthquakes with a magnitude larger than M for the period covered by the earthquake catalogue; a and b are the GR parameters. The number of events with a magnitude larger than M can be provided for shorter periods (for example, per year or day). In this case, $\lambda_{M}$ is also known as the annual or daily seismic activity rate. The computation of the seismic activity rate is needed when PSHA is obtaining the likelihood of at least one event of magnitude higher than M within a time interval t (usually one year for conventional PSHA and one day for TD-PSHA). This probability, named the probability of exceedance, is calculated using Equation (2), assuming that the occurrence of earthquakes follows a Poisson distribution.

(2) $$P\left(M\right)=1-e^{-t\cdot \lambda_{M}}$$

Thus, using the mean annual (or daily) exceedance rate of earthquakes, the probability density function of earthquake recurrence, the probability density function of source-to-site distance, and the probability of exceedance, a given ground motion value conditioned to the magnitude and distance (i.e., the PSHA or TD-PSHA) are computed using the classical Cornell [19] approach.

Therefore, if a direct relationship is established between the radon anomalies in a given seismic region and the temporal changes in the daily seismic activity rate in that area, the TD-PSHA could be computed for any site of interest providing a forecast of the daily probability of exceedance of a given ground motion, which can later be converted to the daily probability of exceedance of a fixed-loss ratio. These results would be very valuable for the current OEF systems.

The aim of this work is to investigate the relationship between radon anomalies and the daily seismic activity rate and develop a methodology that allows estimating the seismic activity rate using only radon measurements. To carry out this study, the earthquake catalogue of the Vrancea region (Romania) has been used to estimate the daily seismic activity rate during a given period, in which radon measurements were also recorded, and obtain a correlation model between both variables.

Previous to any correlation, seasonal periodicities as well as linear trends, which might be due to other non-seismic factors such as meteorological ones (e.g., temperature, pressure, etc.), have been removed [20,21]. After that, the discrete wavelet transform has been used to analyse the radon signal on two different scales: long and short periods.

Additionally, the study carried out reveals certain characteristics that could be used as precursors of earthquakes at different scales: weeks/months in the case of the estimated daily seismic activity rate, and days/weeks in the case of the short-period signal obtained by the wavelet analysis.

The surveying of radon concentration is not only used in earthquake forecasts but also in fracture trace and environment monitoring and is the subject of many environmental programs, especially because radon is considered a natural hazard and the main contributor to lung cancer second to smoking. Thus, earthquake forecasts should benefit from the existence of radon-monitoring networks all over the world, even if their main purpose is different from the seismic ones.

This paper is structured as follow: The area under study and the data recordings are presented in Section 2. After that, a brief description of the different methods used in the analysis of the time series is introduced in Section 3. The paper continues with the analysis and discussion of the results (Section 4) and the main conclusions (Section 5).

## 2. Seismic Settings and Data Acquisition

**Figure 1:**
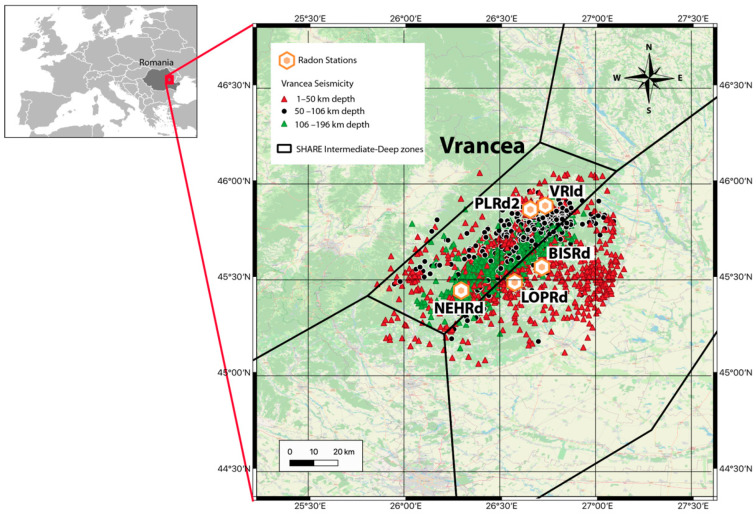
Seismicity in the Vrancea region (Romania) from July 2015 to August 2020. The SHARE intermediate-deep seismic zone [23,24] and the location of the radon stations are also represented.

The Vrancea zone represents one of the most active seismic zones in Europe and is located on the eastern edge of the strongly bent Carpathian arc. The curvature of the Carpathian Mountains is characterised by many faults and intermediate-deep earthquakes. The area has been monitored to highlight the phenomena that precede intensive seismic periods for a short-term forecast. The monitoring equipment has been located according to the geological features of the location (Figure 1). Every station sends information to the NIEP centre (National Institute for Earth Physics from Romania) automatically. Radon monitoring occurs indoors in the air near the ground using Radon Scout PLUS sensors, produced by SARAD, at a sampling rate of 3 h. The seismicity in the area is characterised by shallow seismicity with moderate earthquakes (Mw < 5.6) together with intermediate-depth activity featuring strong earthquakes (Figure 1). More information about the deployed monitoring network can be found in [22].

**Figure 2:**
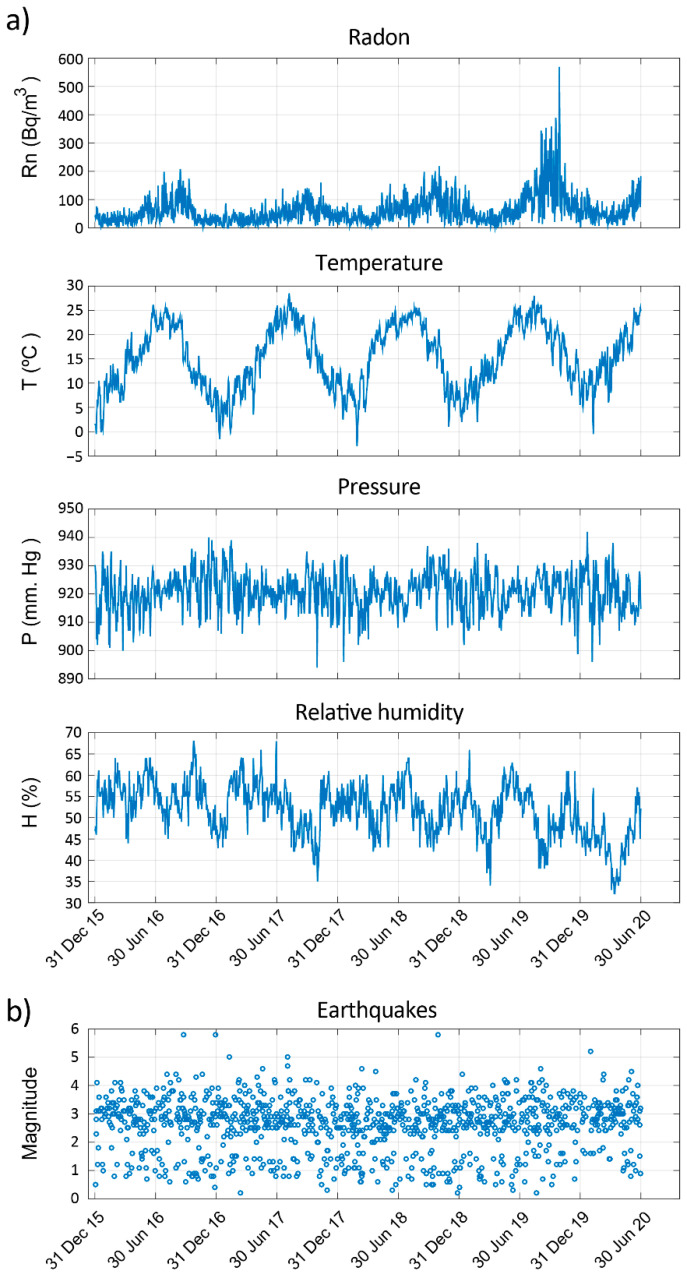
(**a**) Environmental measurements taken at the BISRd station: Radon, temperature, pressure, and relative humidity. (**b**) Earthquakes registered in the Vrancea region between 31 December 2015 and 30 June 2020.

For the present study, the measurements taken at the BISRd station have been used, as it is located in the most central part of the Vrancea zone and covers a longer period of time without gaps, spikes, or corrupted data. The station is installed inside a place made with brick walls. In Figure 2, radon, temperature, pressure, and relative humidity measurements are shown for the BISRd station for the period between 31 December 2015 and 30 June 2020. Additionally, the earthquakes registered during this period are also shown. As can be observed, seismicity in this region occurs almost daily.

According to Figure 1 and Figure 2, a seismic catalogue covering the whole period in which there are radon recordings has been selected. To assure a correct computation of the GR parameters for the first days of the radon measurements, the seismic catalogue starts several months before the starting day of the radon measurements. Hence, the seismicity from 1 July 2015 to 30 September 2020 is used to analyse the correlations between the seismic activity rate and the radon activity. The catalogue is part of the official NIEP catalogue that can be downloaded from http://www.infp.ro/index.php?i=romplus (accessed on 29 April 2022) and it contains 2158 events with a moment magnitude range ($\Delta M_{w}$) between 1.2 and 5.5 and depths from 1.0 to 196 km. There is a mean of 30 events by month. Most of the earthquakes have magnitudes greater than 2.5. Most of the events have a depth greater than 50 km, i.e., 39% are shallow seismicity (1 to 50 km) and 61% are intermediate-deep seismicity (below 50 km depth).

As it can be seen in Figure 1, the earthquakes are found within a maximum distance of 45 km from the BISRd station. Thus, by applying the relationship proposed by Papachristodoulou et al. [9] (i.e., R_E_ ≤ 1.5 × 10^0.43M^), the radon station should record all the radon anomalies due to earthquakes with a magnitude M > 3.5. Hence, the magnitude of completeness for the radon anomalies should be around this minimum value.

## 3. Methods

### 3.1. Discrete Wavelet Transform (DWT)

The discrete wavelet transform (DWT) can be described as a sub-band coding scheme [25] where the signal is iteratively divided into low and high frequencies by applying two quadrature filters plus downsampling. Thus, the signal is decomposed into a finite number of scales or levels. The high-pass filter, known as the wavelet filter, is derived from a basic function called the mother wavelet, meanwhile, the low-pass filter is known as the scaling filter.

The coefficients obtained from the low-pass filters represent the approximation of the signal at different scales. On the other side, the resulting signals from high-pass filters are known as wavelet or detail coefficients and provide the details of the input signal at different scales or resolutions.

In this way, any signal can be analysed at different time-frequency resolutions by properly selecting the approximation and wavelet coefficients. The contribution of one or several of these coefficients to the original signal can be analysed by zeroing the rest of the coefficients and then reconstructing the signal through the inverse transform. Therefore, a wavelet band-pass filter can be accomplished (e.g., [26,27]).

In the present work, the input time series is split into two signals. The low-frequency behaviour is extracted by selecting the approximation coefficients of the maximum scale. Meanwhile, the high-frequency components or details are obtained with the contribution of all the other wavelet coefficients.

More details of the discrete wavelet analysis can be found in [28,29,30].

### 3.2. Cross-Wavelet Transform (XWT) and Wavelet Coherence (WTC)

The cross-wavelet transform (XWT) and the wavelet transform coherence (WTC) allow investigating relationships between two signals along time and frequency and determining common behaviours between them.

The study of interrelation between pairs of time-domain signals can be performed by the application of the XWT, which is defined as the product of the respective wavelet transforms. As a result, the cross-wavelet power and the local relative phase between both signals in the time–frequency space are obtained.

In the other way, the WTC analyses the coherence and phase lag between two time series as a function of both time and frequency. The higher the coherence between the signals, the higher the relation in terms of frequency and phase. Areas with high common power between signals are identified.

More details about the XWT and WTC can be found in [31,32].

### 3.3. Short-Time Correlation Analysis (STC)

Correlation analysis is a statistical method used to assess the relationship between two different signals. It can be quantified through the Pearson correlation coefficient, which takes an absolute value between 0 (no correlation) and 1 (perfect correlation). If both signals are well-correlated, a linear regression analysis can be carried out in order to estimate the mathematical equation that relates both signals.

In the case of stationary signals, the correlation analysis works correctly. However, for non-stationary signals and especially for relatively long time series, the correlation analysis does not provide clear insights into the possible relationship.

In this work, a new approach is proposed and implemented in order to study the possible correlations along the time.

For this, the signals are analysed day by day, applying a time window centred on the selected day and estimating the best correlation coefficients. For each day, any possible lag that may exist between both windows is also taken into account. Thus, the window applied to one of the signals is centered on the selected day, *dselect*; in the other time series, 2 × *lag* + 1 windows are analysed, whose centres will go from the *dselect* − *lag* to the *dselect* + *lag*. Once the windows that provide the best correlation coefficient for the selected day have been determined, the best linear fit is obtained.

At the end of the time series analysis, the slopes and y-intercepts associated with the best linear fit obtained for each of the days are averaged. For this calculation, only the days with a correlation coefficient higher than 0.9 will be used.

### 3.4. Similarity Analysis

In order to evaluate the obtained results, three different metrics are used to investigate the resemblance or deviation between the expected and the predicted signals. Concretely, the root mean square error (RMSE), the correlation coefficient (R^2^), and the similarity coefficient (S) will be applied for every time window of analysis (see Section 3.3).

The RMSE is used to measure the degree of the standard deviation between the observed and the predicted signals. The smaller the RMSE value, the closer the prediction values to the observation values.

The value of R^2^ reflects the linear correlation between the prediction and the observation time series. Its value ranges between 0 and 1. For the correlation coefficient, less than 0.3 is considered no correlation, and 0.3 to 0.8 is considered a weak correlation [33].

Finally, the similarity coefficient between two signals is a measurement of the consistency of the respective time series. It takes a value between zero and one and it is equal to one only if the waveforms of both signals are completely the same in shape and amplitude [34]. In practice, the value will reach its maximum.

## 4. Results and Discussions

### 4.1. Seismic Activity Rate

The seismic activity rate is computed using the methodology proposed by Gulia et al. [35]. Following this methodology, a continuous GR parameter time series is obtained from devoid artifacts [36,37]. A fixed number of events (100 events) is chosen for each window of analysis and they are moved through the catalogue event by event, thus exploring the full range of variability in the data [35]. The correct assessment of the completeness magnitude is critical for the correct estimation of the GR parameters; therefore, the maximum curvature method is used [36]. The GR parameters are usually plotted at the beginning, middle, or end of the time window that they represent. Following Tormann et al. [36], the values are assigned to the end of the time window as it is the most sensible to physically understand the resolved signals.

The software ZMAP [38,39] is used for computing the temporal evolution of the GR parameters, a and b, whose values allow computing the temporal evolution of the seismic activity rate ($\lambda_{M}$) for a given threshold magnitude (M). Finally, $\lambda_{M}$ is resampled in such a way that it has a value per day, which provides the daily seismic activity rate.

**Figure 3:**
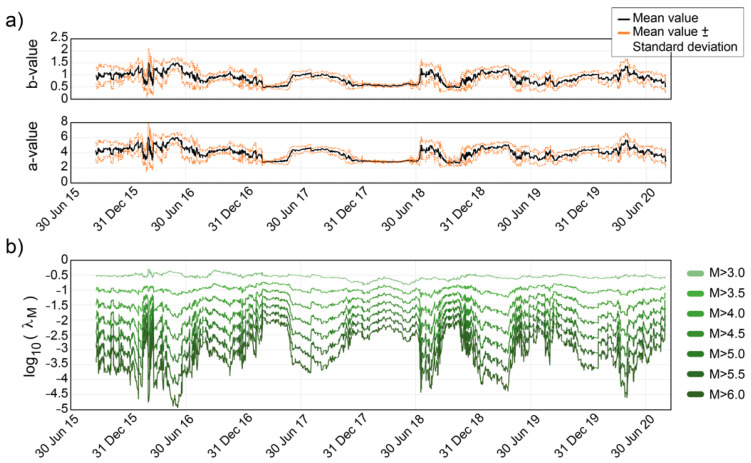
(**a**) Temporal evolution of the GR parameters computed for the Vrancea region. (**b**) $\log_{10}\left(\lambda_{M}\right)$ for different threshold magnitudes.

Thus, in the present work, the continuous GR parameter time series, a and b, have been computed from the Vrancea full catalogue (from July 2015 to September 2020) using the methodology explained earlier (Figure 3a). Subsequently, these values have been used to obtain $\log_{10}\left(\lambda_{M}\right)$ for different threshold magnitudes, M (Figure 3b).

In Figure 3b, it can be observed how the curves obtained for the different magnitudes follow a similar behaviour. As the magnitude decreases, the mean value of $\log_{10}\left(\lambda_{M}\right)$ changes and its range of variation becomes smaller and smaller, reaching a practically flat curve for M ≥ 3.0.

A linear regression analysis between the curve obtained for M > 6.0 and the other curves, provides the mathematical expression that relates them. In this way, once the daily seismic activity rate is obtained for M > 6.0, i.e., $\log_{10}\left(\lambda_{6.0}\right)$, it can be estimated for any other magnitude by applying Equation (3):

(3) $$\log_{10}\left(\lambda_{M}\right)=f_{1}\left(M\right)\cdot \log_{10}\left(\lambda_{6.0}\right)+f_{2}\left(M\right)$$

where $f_{1}\left(M\right)$ and $f_{2}\left(M\right)$ are the slope and the y-intercept, respectively, of the linear fitting. These values are different for each magnitude, hence their dependence on the variable M is indicated.

**Figure 4:**
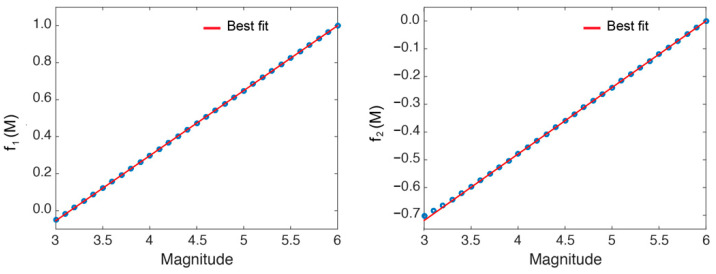
Linear regression analysis between the curve, $\log_{10}\left(\lambda_{6.0}\right)$, and the other ones, $\log_{10}\left(\lambda_{M}\right)$. $f_{1}\left(M\right)$ and $f_{2}\left(M\right)$ are the obtained slopes and y-intercepts, respectively. The best fit (red line) is also shown.

In Figure 4, the $f_{1}\left(M\right)$ and $f_{2}\left(M\right)$ values obtained for different magnitudes are shown. It can be observed how the slope and the y-intercept estimated for the different magnitudes between 3.0 and 6.0 follow a linear behaviour, at least from 3.5 onwards.

A linear regression analysis of these values returns the following relations Equation (4):

(4) $$f_{1}\left(M\right)=0.35\cdot M-1.11\;f_{2}\left(M\right)=0.24\cdot M-1.44$$

Attending to the results shown in Figure 3b and Figure 4, the magnitude of completeness might be established around 3.5.

### 4.2. Meteorological Effects

Several authors [9,20,21,40,41,42,43,44,45] have pointed out the importance of removing the effects of meteorological parameters, as temperature can feature multiple seasonality on the radon temporal series.

In Figure 2, the time series of radon, temperature, pressure, and relative humidity are shown. At first sight, the temperature series is the one that presents a clear seasonal behaviour.

Initially, the relationship between the radon measurements and the local climate variables has been measured with the Pearson’s correlation coefficient. However, long non-stationary time series such as these are unlikely to obtain good results when they are compared as a whole.

**Figure 5:**
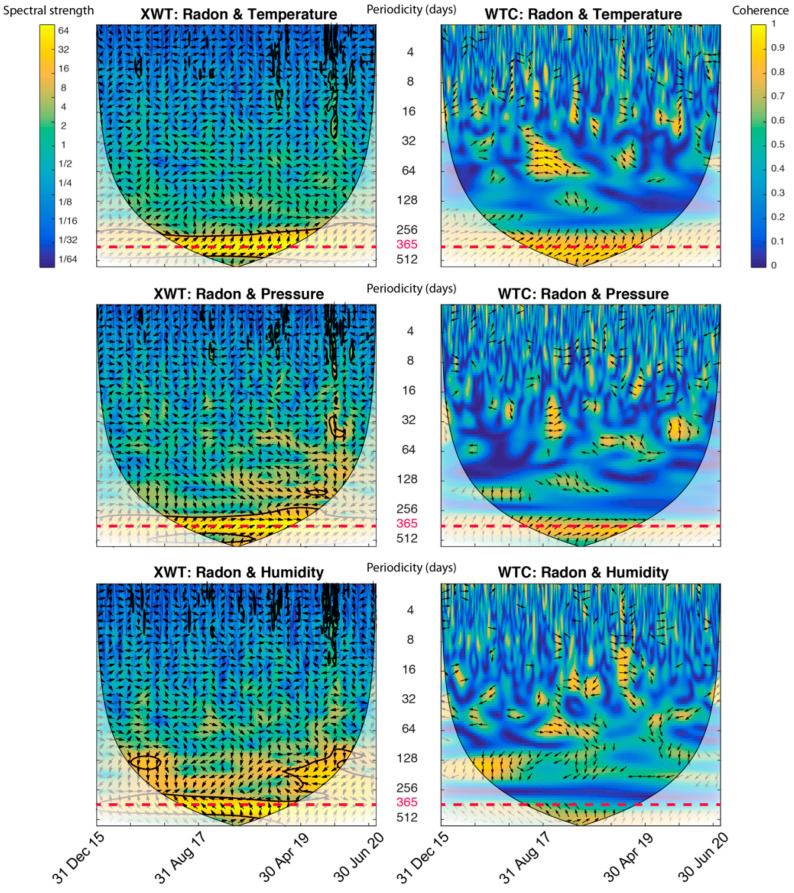
XWT and WTC performed in pair of signals: Radon—temperature; radon—pressure; and radon—relative humidity. The bottom axis is time. Spectral strength is shown by colours ranging from blue (weak) to yellow (strong). The central vertical axis indicates periodicities (days). The relative phase relationship is shown as arrows (with in-phase pointing right, anti-phase pointing left, and one signal leading the other by 90° pointing straight down) and can be also interpreted as a lead/lag of one signal in relation to the other.

Alternatively, the XWT and WTC have been used to explore the possible interdependencies between these variables (Figure 5). The XWT and the WTC between pairs of signals have been implemented with software provided by Grinsted et al. [31], available as a MATLAB software package. The statistical significance level of the WTC is determined by Monte Carlo methods [46]. Edge effects caused by discontinuities at endpoints are evaluated by the Cone of Influence (COI) [47].

In Figure 5, the periodicity at the 365-day band is identified when performing the XWT and WTC. The spectral strength and coherence range from dark blue (weak) to yellow (strong). For all the plots, the cone of influence (curved lines) determines the area where the edge effects cannot be ignored. The border distortion is usually caused by insufficient data points both at the beginning and end of finite-duration signals. The edge effect increases with scale and reduces the effective length of the analysed data series.

Thus, the results obtained with the XWT show how the radon is well correlated with the other three variables (temperature, pressure and relative humidity) for periods of 365 days.

From the point of view of the coherence between the signals, the WTC shows also its maximum amplitude at the 365-day nark, especially when we relate radon to temperature or pressure, with coherence values higher than 0.8.

In the case of the radon–temperature relation, it has been frequently observed in other works [48,49,50,51]. In the present study, the XWT and WTC plots show how the direction of the arrows remains constant throughout the 1-year period, which indicates that the phase difference between these radon and temperature variables is constant, or almost constant, over time. It is important to note that this phase difference is dependent on the sites’ locations and therefore strongly controlled by local factors [52].

Thus, the occurrence of the 1-year cycle in the radon and other environmental measurements suggests that these observations could be related in some way to a common causative factor.

From the previous analysis, it can be deduced that the radon series includes oscillations of 1-year periods, which are due to other external factors, but cannot be attributed to the seismicity of the area. Therefore, it is crucial to eliminate this seasonal behavior beforehand.

**Figure 6:**
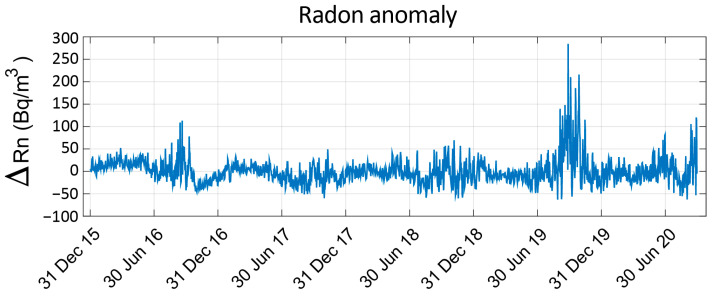
Radon anomaly after seasonal and linear trend removal.

In the present work, the 1-year oscillations, as well as any possible linear trends, have been identified by using cubic-spline interpolation [27]. In Figure 6, the radon anomaly that corresponds to the subtraction of the seasonal behaviour from the radon time series is shown.

### 4.3. Data Preprocessing

The radon anomaly has been studied at two different scale ranges through the DWT application.

Concretely, the DWT has been applied to the radon signal (obtained from Section 4.2) and the daily seismic activity rate (obtained from Section 4.1). As the wavelet mother, the Daubechies 10 has been selected, which experimentally has been considered appropriate for the study of other microclimatic signals [53]. A maximum level of decomposition equal to 6 has been selected.

On the one hand, the signals corresponding to the approximation coefficients have been selected, which implies the study of periodicities over 64 days (maximum level, 6). These signals will be used to study the relationship between the radon anomaly and the daily seismic activity rate.

On the other hand, the sum of the wavelet coefficients of the radon anomaly has also been selected to investigate small periodicities below 64 days. In this case, deviations from the mean value might be related to incoming earthquakes.

**Figure 7:**
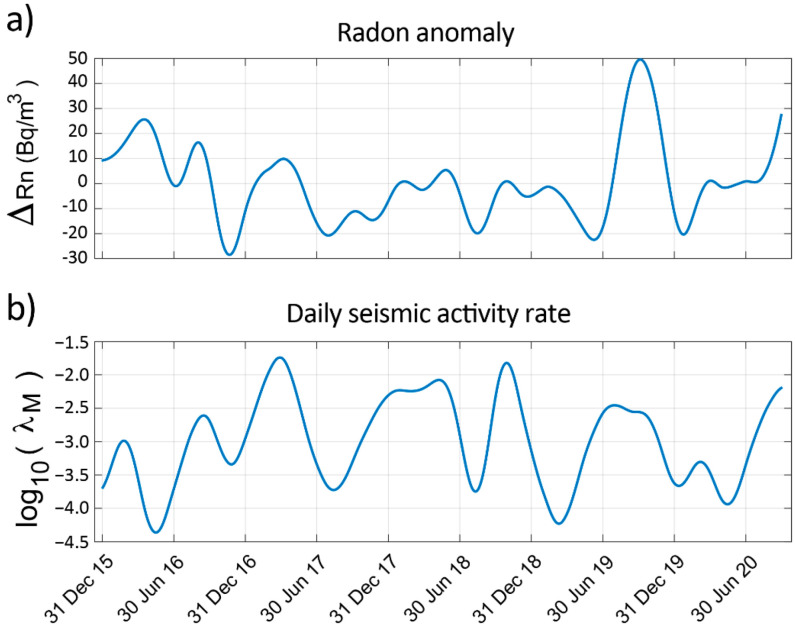
Approximation coefficients from the DWT analysis: (**a**) Radon anomaly and (**b**) daily seismic activity rate (M > 6.0).

The DWT analysis has been carried out using the software provided by Galiana-Merino et al. [27]. In Figure 7, the obtained approximation coefficients are shown.

### 4.4. Radon and Seismic Activity Rate Relation

The STC approach explained in Section 3.3 has been applied to the approximation coefficients in order to investigate possible correlations between radon and the daily seismic activity rate.

For each window of analysis, the best linear adjustment is calculated. After that, the median value (slope and y-intercept) is selected for the whole time series. It is not intended to indicate that there is a direct linear relationship between both variables. The objective is to be able to roughly estimate the behaviours (rise and fall intervals, peaks and troughs) of the daily seismic activity rate from the radon measurements.

**Figure 8:**
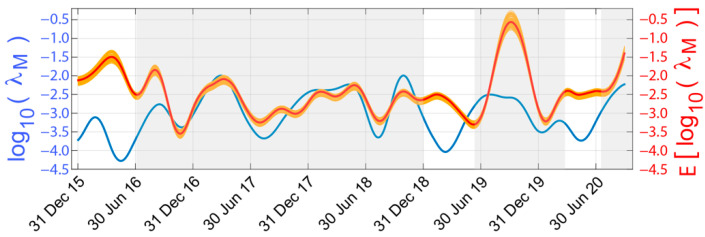
Comparison between the expected $\log_{10}\left(\lambda_{M}\right)$ (blue line); the 100 predicted curves from the 2-year interval analysis (orange lines); and the predicted curve using the median values, $E\left[\log_{10}\left(\lambda_{M}\right)\right]$, (red line). The grey shaded areas indicate intervals with higher similarity.

In order to study the robustness of the proposed approach, the STC analysis has been applied to 100 2-year intervals, arbitrarily selected from the entire series. In Figure 8, the comparison between the expected daily seismic activity rate, i.e., $\log_{10}\left(\lambda_{M}\right)$, and the predicted one from the radon anomaly, i.e., $E\left[\log_{10}\left(\lambda_{M}\right)\right]$, is shown.

The orange curves are the predicted signals obtained from the analysis of the 100 2-year intervals arbitrarily selected. As observed, they present a very low dispersion around the median value (red curve), which is indicative of the robustness of the obtained results for these time series.

The grey shaded areas try to draw attention to the time intervals in which the predicted curve follows the expected behaviour (increasing/decreasing intervals, peaks and troughs).

Thus, the approximate relationship between both variables using the median values of the slope and the y-intercept is expressed for the site under study as Equation (5):

(5) $$E\left[\log_{10}\left(\lambda_{M}\right)\right]=0.0388\;\frac{m^{3}}{Bq}\Delta \cdot \mathrm{Rn}-2.4634$$

where ΔRn corresponds to the approximation coefficients obtained in Section 4.3 for the radon anomaly. For the study site, this relation provides insights into the behaviour of the daily seismic activity rate from the radon measurements. We must remember that this is not an exact relationship between both signals as a radon anomaly might be affected by other additional factors.

**Figure 9:**
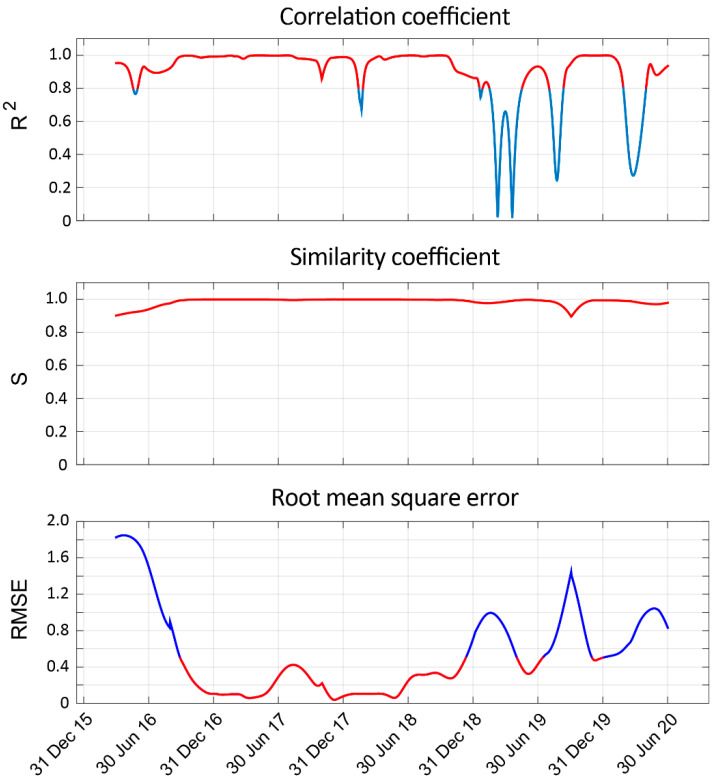
Correlation coefficient, similarity coefficient, and root mean square error for the entire time series. The intervals with the best results are shown in red.

As explained in Section 3.4, three different metrics have been used to evaluate the performance of the STC approach: the correlation coefficient, the similarity coefficient, and the root mean square error. In Figure 9, the results estimated for each day of analysis are shown. In this case, the complete time series have been analysed.

In the case of R^2^, values higher than 0.8 indicate a very good correlation. As for S, the closer the value to 1, the greater the degree of similarity. Finally, the value of RMSE is recommended to be below 0.5. Attending to these considerations, the curves shown in Figure 9 have been plotted in red for those intervals in which these criteria are met.

In the case of the RMSE values, it can be seen that the largest deviations from the expected results occur mainly at the beginning and end of the time series, which might be associated with some border effects due to the lack of previous (or subsequent) data.

Important deviations also appear around the interval from 30 June 2019 to 31 December 2019, especially for the R^2^ and RMSE values. In this time interval, the radon signal presents a peak of relatively large amplitude compared to the other peaks, which must be due to other as-yet-unknown external factors.

Regarding the S value, it is important to highlight how it remains above 0.9 for all the time series.

### 4.5. Application to Forecasting Studies

From the analysis carried out in the previous section, we can observe that the behaviour of the daily seismic activity rate can be estimated roughly using radon measurements. Applying Equations (3)–(5), the correlation between radon anomaly and the daily seismic activity rate can be estimated for any magnitude above the magnitude of completeness. Once estimated $\log_{10}\left(\lambda_{M}\right)$, the probability of exceedance can also be obtained.

Each day, a radon series of 2 years prior to the current day could be analysed and an estimate of the possible daily seismic activity rate could be made. Thus, increases in the seismicity of the analysed site could be detected through radon measurements.

**Figure 10:**
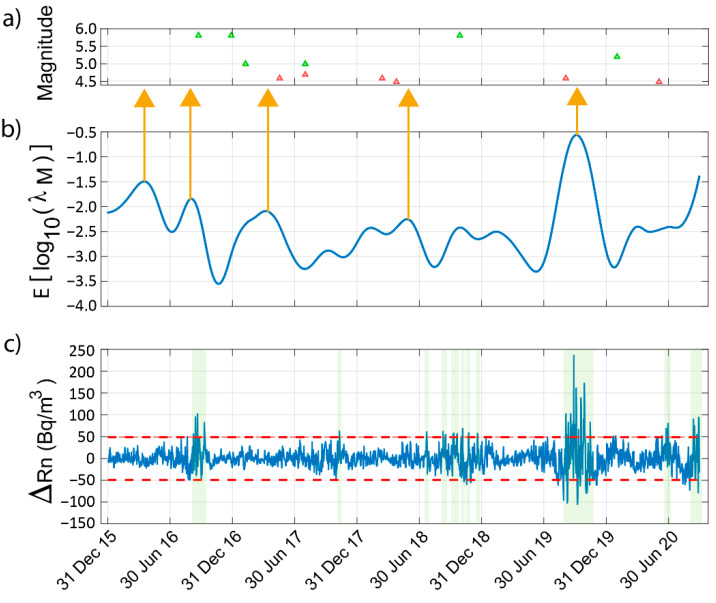
(**a**) Earthquakes of magnitude greater than 4.5 occurring in the Vrancea region, (**b**) $\log_{10}\left(\lambda_{m}\right)$ estimated from the radon measurements. (**c**) Sum of the wavelet coefficients of the radon anomaly (see Section 4.3). The dashed red lines indicate ±2 the standard deviation.

In Figure 10, the estimated daily seismic activity rate (Figure 10b) is compared with the sequence of earthquakes of magnitudes greater than 4.5 that occurred in the Vrancea region (Figure 10a). Considering the seismicity in the region under study (Figure 2a), we can see that most of the registered earthquakes have a magnitude below 4.5 and occur daily. Thus, in order to identify some characteristics in the seismic activity rate that might be indicative of incoming stronger earthquakes (according to the magnitudes in the area), we have chosen 4.5 as the magnitude threshold since above it there are only a dozen events in the analysed period.

If we compare the estimated $\log_{10}\left(\lambda_{M}\right)$ with the sequence of earthquakes of magnitudes greater than 4.5 that occurred in the Vrancea region, then we can observe how earthquakes of magnitudes M ≥ 4.5 have been preceded by increases in seismicity and local maximums, detected sometimes several weeks/months prior (see vertical orange arrows). Thus, it might be used as an input for a possible design of a forecasting system for the region.

In Figure 10c, the sum of the wavelet coefficients of the radon anomaly are also shown (see Section 4.3). In addition, two dashed red lines are included, which indicate ±2 the standard deviation. According to other authors (e.g., [3,8,12,13,14,15]), a positive deviation that exceeds the mean radon level by more than twice the standard deviation might be indicative of incoming earthquakes. In the present study some deviations of this type can be observed that occur a few days/weeks before some of the earthquakes with magnitudes above 5.0 (see green shadow areas).

As a conclusion, both types of information could be used as input for computing the TD-PSHA in the region under study. Concretely, when computing the TD-PSHA, a logic tree can be drawn in such a way that a branch represents the computation of $\lambda_{M}$ using conventional methods and other branches using the radon measurements, allowing a better representation of the uncertainties when providing OEF metrics (hazard gain, loss gain) to stakeholders.

## 5. Conclusions

In this work, the relationship between the radon time series and the daily seismic activity rate has been investigated and a new methodology that allows estimating in advance the seismic activity rate using only radon measurements has been developed.

The study carried out is based on the fact that daily radon release is related to the cumulative effect of daily seismicity in the area of influence of the recording station, and not only to the effect that a single earthquake can produce.

The proposed methodology has been tested in the Vrancea region (Romania). The earthquake catalogue used comprises a given period in which radon measurements were also available, that is, from January 2016 to September 2020.

The daily seismic activity rate for a given threshold magnitude has been computed using the temporal evolution of the GR parameters, a and b, which were previously estimated following the methodology of Gulia et al. [35].

The relationship between radon and other climate variables has been also studied by wavelet analysis. Periodicities of 365 days have been identified and subsequently removed from the radon time series. After that, the DWT has been applied to split the radon signal into two components: approximation coefficients (long periods) and the sum of the wavelet coefficients (short periods). A short-time correlation analysis has been subsequently applied to the approximation coefficients of the radon signal and the daily seismic activity rate, obtaining a linear relationship between both variables. Although there are other external factors that can affect the radon signal, this linear approximation allows an estimation of the daily seismic activity rate from only the radon series and provides insights into a possible increase/decrease in seismic activity in the region under study.

Finally, the comparison of the estimated daily seismic activity rate and the wavelet coefficients of the radon signal with the sequence of earthquakes with a magnitude higher than 4.5, reveals certain characteristics of both signals that could be used as precursors of earthquakes at different scales: weeks/months in the case of the seismic activity, and days/weeks in the case of the wavelet coefficients.

The forecasting of the daily seismic activity rate from radon anomalies could become an important tool for the development of new time-dependent probabilistic seismic hazard analyses. In any case, additional studies should be carried out for other regions and radon stations.

## Data Availability

All data included in the manuscript are available upon request by contacting the corresponding author.
